# Innovative Approaches to Combat Duck Viral Hepatitis: Dual-Specific Anti-DHAV-1 and DHAV-3 Yolk Antibodies

**DOI:** 10.3390/vaccines13020154

**Published:** 2025-02-02

**Authors:** Siqi Lei, Yuanhe Yang, Chengchen Zhao, Anguo Liu, Pingli He

**Affiliations:** State Key Laboratory of Animal Nutrition and Feeding, Frontiers Science Center for Molecular Design Breeding (MOE), China Agricultural University, Beijing 100193, China; leiske2000@163.com (S.L.); yyh8695@163.com (Y.Y.); zcc15872612567@outlook.com (C.Z.); angelo524@foxmail.com (A.L.)

**Keywords:** duck viral hepatitis, duck hepatitis A virus, egg yolk antibody, treatment, antioxidation

## Abstract

**Background and Objectives:** Duck viral hepatitis (DVH), a highly contagious and acutely fatal avian disease, is characterized by convulsions, acute death, liver swelling, and hemorrhage, leading to substantial losses in the duck industry. However, there is no efficient prevention and control method for DHV infection. Duck hepatitis A virus (DHAV) is one of the primary pathogens responsible for DVH. **Methods**: In this study, we prepared a highly effective anti-DHAV IgY antibody by immunizing Hy-Line Brown laying hens at the peak of egg production. **Results and Conclusions**: The neutralization index of this antibody was found to be up to 38.90 (DHAV-1 QYD strain) and 141.25 (DHAV-3 GY strain) in vitro. The antibody also exhibited effective prophylactic effects in a model of hepatic inflammation following the viral challenge of ducklings, with a dose of 0.5 mL per duckling (containing 64 mg/mL of IgY) significantly reducing DHAV-related mortality by 66%, providing substantial protection against the infection. Furthermore, it effectively alleviated oxidative damage caused by DHAV in the ducklings. The results of this study indicate that IgY has the potential for treating DHAV infection; it also provides a new way for the treatment of poultry diseases with specific antibodies.

## 1. Introduction

Duck viral hepatitis (DVH) is an acute and highly lethal contagious disease of ducklings, which leads to mortality rates as high as 95% in ducklings. Duck viral hepatitis is caused primarily by the duck hepatitis A virus (DHAV). As one of the major pathogens responsible for DVH, DHAV is categorized into three distinct genotypes: DHAV-1 (classical genotype A), DHAV-2 (genotype B), and DHAV-3 (genotype C). The two main genotypes are DHAV-1 and DHAV-3 in China. These genotypes primarily affect the liver and spleen in ducklings which leads to liver and spleen enlargement, and hepatic hemorrhage [1]. The widespread prevalence of DHAV, particularly in the absence of reliable commercial vaccines and treatments, poses a significant threat to the duck farming industry. In regions with high farm densities, the rapid spread of DHAV following an outbreak can be challenging to control. Consequently, DHAV has inflicted significant economic losses on the global duck industry, with a particularly severe impact in Asia. In China, from 2001 to 2021, the incidence rate of DHAV was about 12% [2]. Vaccination is the primary method used to control DHAV. While the use of live DHAV-1 vaccine has led to a decrease in DHAV-1 infections since 2013, the vaccine has not completely controlled the incidence of DHAV-1. Outbreaks of DHAV still occur in many ducklings vaccinated with the attenuated DHAV vaccine [3]. Moreover, DHAV-3 has now emerged as a significant cause of DHAV infection but the approved live DHAV-3 vaccine has not been used widely [4]. Researchers have increasingly focused on protective effects of various herbal extracts, such as flavonoids and polysaccharides [5,6,7,8], although their efficacy remains questionable.

Recently, egg yolk antibody (IgY) has demonstrated efficacy in preventing livestock disease, such as avian influenza virus (AIV) [9], newcastle disease virus (NDV) [10], duck adenovirus (DAV) [11], goose astrovirus (GAstV) [12], rotavirus (RV) [13], porcine epidemic diarrhea virus (PEDV) [14], infectious bursal disease virus (IBDV) [15], and dengue virus (DENV) [16]. Immunoglobulin Y, the predominant antibody in birds, reptiles, and amphibians, is transferred to the yolk through maternal blood and provides innate passive immune protection in the embryo and hatched chick. Immunoglobulin Y may act through several mechanisms to reduce pathogen levels and disease severity in the host, by providing passive immunity against viruses or bacteria binding to antigens on pathogen surfaces to prevent their attachment to host cells, by accelerating antigen processing and presentation through Fc receptor (FcR)-mediated endocytosis in the case of birds, and by activating the complement system. It can also serve as an adjunct to vaccination where such has not adequately elicited a broadly protective immune response or during the early post-vaccination period [17,18]. In addition, IgY technology offers several advantages over other antibody sources, because it allows non-invasive collection, is low cost, produces high antibody yields, possesses unique structural properties, and is thermally and pH stable. Additional benefits of the IgY antibody include reduced immunogenicity, lower risk of immune-related side effects, and interaction with the mammalian complement system to enhance the accuracy of immunological assays by minimizing non-specific reactions [17,19,20]. Using hens to produce IgY against DHAV-1 and DHAV-3 is advantageous due to their cost-effectiveness, high antibody yield, and ethical benefits, as it avoids directly infecting ducks. Therefore, utilizing IgY for disease prevention and control showcases the application and advancement of biotechnology in animal husbandry and meets the international market’s demand for healthy and safe food.

In this study, the anti-DHAV IgY was prepared using laying hens immunized six times with inactivated DHAV. Our objective was to present a novel and effective strategy for prevention and control of DHAV infections.

## 2. Materials and Methods

### 2.1. Management and Immunization Protocol for Laying Hens

Eighteen 30-week-old Hy-Line Brown laying hens were kept in 3-tiered cages and randomly assigned to one of three immunization treatment groups, with 3 replicates per group and 2 hens per replicate. After a one-week pre-feeding period, laying hens were immunized. Group A (GA) was vaccinated with the Duck Viral Hepatitis Attenuated Vaccine, Living (Strain CH60), from Harbin Pharmaceutical Group Bio-vaccine Co., Ltd., Harbin, China. Group B (GB) received the Duck Viral Hepatitis Bivalent (Serotype 1 + Serotype 3) Vaccine, Inactivated (Strain YB3 + Strain GD), from Qingdao Yebio Bioengineering Co., Ltd., Qingdao, China. The control group (GCON) was injected with an equivalent volume of physiological saline. Each hen received an initial injection at week 0, followed by booster immunizations administered at weeks 4, 8, 12, 16, and 20 to maintain antibody levels [21]. Each dose of 1 mL was injected into the pectoral muscle at multiple sites. Throughout the experimental period, egg yolks were continuously collected, purified, and antibody titers were monitored at weeks 5, 9, 13, 17, 21, 29, 37, 45, 49, 53, 57, and 61.

### 2.2. Extraction and Purification of IgY

For the current experiment, IgY was prepared using the polyethylene glycol precipitation method [22]. In the initial step, highly immunized eggs were cleaned, sterilized with benzalkonium bromide solution (Vujeen, Hebei, China), wiped with 75% alcohol, and cracked to isolate the antibody-rich yolks. Antibodies were then extracted from the yolks using the polyethylene glycol precipitation technique. This involved mixing the yolks with PBS (phosphate-buffered saline, PBS) (Solarbio, Beijing, China), adding 3.5% (*m*/*v*) PEG-6000 (Solarbio, Beijing, China) [23,23], centrifuging the mixture at 12,000 rpm for 20 min using a Sigma 3-30KS centrifuge with a fixed-angle rotor 12150 (Sigma Laborzentrifugen GmbH, Osterode, Germany), filtering the supernatant, and re-dissolving the precipitate in PBS through a series of steps. The final solution was dialyzed, filtered, and stored at 4 °C. Subsequent steps included determining the protein concentration, analyzing the solution with SDS-PAGE electrophoresis, and storing the validated antibody at −20 °C.

### 2.3. Determination of Antibody Titer by ELISA

Enzyme-linked immunosorbent assay (ELISA) was regularly employed to monitor the dynamic changes in IgY titers. Briefly, the DHAV-1 and DHAV-3 viral solutions were diluted to 100 50% embryonic lethal dose (ELD_50_)/mL with CBS (Carbonate-Bicarbonate Buffer Solution, CBS) (Solarbio, Beijing, China) solution. Subsequently, the diluted solution was added to a 96-well plate and incubated at 4 °C for 12 h. After five washes with PBST (phosphate-buffered saline with Tween 20, PBST) (Solarbio, Beijing, China), the antibody to be tested, prepared at an initial concentration of 2 mg/mL and diluted 1:1000, was introduced and incubated at 37 °C for 1 h. Following this incubation, the antibody underwent five washes with PBST, followed by the addition of a rabbit anti-chicken secondary antibody. The plate was then incubated for 1 h at 37 °C. After washing, 3,3′,5,5′-tetramethylbenzidine (TMB) (Solarbio, Beijing, China) color developer was added and allowed to react for 5 min. The reaction was terminated with a stop solution, and the absorbance was measured at 450 nm using a Synergy 4 microplate reader (BioTek, Winooski, VT, USA).

### 2.4. Determination of IgY Neutralization Index by Virus Neutralization Test

The DHAV-1 QYD strain (GenBank sequence number on NCBI: GU066825.1) and the DHAV-3 GY strain (GenBank sequence number on NCBI: EU352805.2) were kindly provided by the experimental group of Prof. Zhang Dabing at China Agricultural University. Eight-day-old embryonated duck eggs were sterilized with alcohol under aseptic conditions. A range of concentrations of the DHAV were mixed with IgY and incubated at 37 °C for 1 h to facilitate antibody-virus binding. The pre-mixed virus-antibody solution (200 µL, consisting of 100 µL virus solution and 100 µL IgY) was then introduced into the duck embryos, which were placed in a thermostatic incubator. Regular assessments of the embryos were conducted every 12 h to document mortality rates and signs of infection in each concentration group. A blank control group (injected with physiological saline) was also set up, following the same procedures as the experimental groups. The neutralization index (NI) was calculated by comparing the viral neutralizing titers of the original virus (10^6.33^ ELD_50_/mL for DHAV-1 and 10^7^ ELD_50_/mL for DHAV-3) with those of the virus neutralized for one hour with IgY antibodies, using the virus neutralization (VN) method. The titers were determined based on the protection rate results, using the Reed–Muench method.

### 2.5. Prophylactic Effect of IgY in Model of Duck Hepatitis Virus Infection

#### 2.5.1. Experimental Protocol

Two hundred and twenty-five 1-day-old Cherry Valley ducks were randomly assigned to one of three treatment groups, with 75 ducks per group. In each group, 15 ducks were designated for sampling and slaughter and were excluded from mortality analysis. Mortality was therefore assessed with 60 replicates per group, with each replicate consisting of one duck. All ducklings were raised under identical management and sanitary procedures, and were fed a basic diet that satisfied nutritional requirements in the NY/T 2122–2012 standard. Throughout the experiment, room temperature was maintained at 30 ± 2 °C and humidity at 70 ± 10%. The photoperiod ranged from 12 to 16 h per day, with a light intensity of 16 Lux. The ducklings were housed in a negative pressure isolation unit within the laboratory animal facility.

The negative control group (Neg CON) was neither infected with DHAV-1 and DHAV-3 nor treated with IgY throughout the experiment. The positive control group (Pos CON) was infected with DHAV-1 and DHAV-3 but did not receive IgY treatment. The prophylactic treatment group (IgY) was also infected with DHAV-1 and DHAV-3 and received intramuscular injections of IgY. At one day of age, the IgY group received an intramuscular injection of 0.5 mL of IgY solution (64 mg/mL) into the thigh muscle, while both the Neg CON and Pos CON groups were injected with PBS. This treatment continued for four days. At two days of age, the Pos CON and IgY groups were injected intramuscularly in the thigh with 0.4 mL of a 100-fold dilution of a 1:1 mixture of DHAV-1 and DHAV-3 viral solutions (DHAV-1 titer: 10^6.33^ ELD_50_/mL; DHAV-3 titer: 10^7^ ELD_50_/mL), while the Neg CON group was injected with physiological saline.

At the early (4 hpi(hours post-infection, hpi), intermediate (8 hpi), and late phases (36 hpi), 5 ducklings were randomly selected for liver tissue and blood sample collection. The ducklings were euthanized with an overdose of sodium pentobarbital (120 mg/kg). Blood samples were left to clot at room temperature for 20 min, then centrifuged at 3000 rpm for 15 min at 4 °C to obtain serum, which was stored at −80 °C for future analysis. Liver samples were collected and fixed in 4% paraformaldehyde, then stored at room temperature.

#### 2.5.2. Mortality and Survival Rates

The mortality dynamics in each group were recorded every 6 h (every 3 h during the peak mortality period) post-challenge. The survival rate of each group was calculated until no further deaths were observed. Dead ducklings were disposed of following biosafety protocols.

#### 2.5.3. Histopathological Examination

The liver tissue of ducklings at 48 h post-treatment was dehydrated, cleared, and embedded in paraffin. Continuous sections approximately 5 μm in thickness were prepared using a microtome, followed by dewaxing and staining with hematoxylin and eosin (H&E). The prepared tissue sections were examined under a microscope (Olympus BX51, Olympus Corporation, Tokyo, Japan) to observe the pathological changes in the liver across different treatment groups. The degree of liver damage, including hepatocellular fatty degeneration, inflammatory cell infiltration, and congestion, was assessed and scored based on the following grading system: 0 = none, 1 = very mild, 2 = mild, 3 = moderate, and 4 = severe.

#### 2.5.4. Assessment of Serum Biochemical Parameters

Serum levels of alanine aminotransferase (ALT), aspartate aminotransferase (AST), alkaline phosphatase (ALP), total protein (TP), and albumin (ALB) were measured using enzyme-colorimetric assays with Module Assembly Type Automatic Analyzer Model 7600 Series (Hitachi, Japan). The serum globulin (GLB) level was calculated by subtracting the ALB level from the TP level. The albumin/globulin (A/G) ratio was calculated as the ratio of ALB to GLB.

#### 2.5.5. Indexes of Oxidative Stress

Serum levels of malondialdehyde (MDA), superoxide dismutase (SOD), catalase (CAT), glutathione peroxidase (GSH-Px), and total antioxidant capacity (T-AOC) were determined using commercial assay kits (Nanjing Jiancheng Bioengineering Institute, Nanjing, China).

### 2.6. Statistical Analysis

All data are presented as the mean and 95% confidence interval (CI), with the experimental unit defined as the individual duckling. A probability (*p*) value of less than 0.05 (*p* < 0.05) was considered statistically significant. Biochemical and antioxidant parameters were analyzed using one-way ANOVA in SPSS software (version 27.0; SPSS Inc., Chicago, IL, USA). The significance of the mean values was evaluated using Tukey’s multiple comparison test. Additionally, changes in antibody titers and survival rate data were analyzed using repeated-measure ANOVA. Results were considered statistically significant at *p* < 0.05.

## 3. Results

### 3.1. IgY Titer Evaluation by ELISA

**Figure 1:**
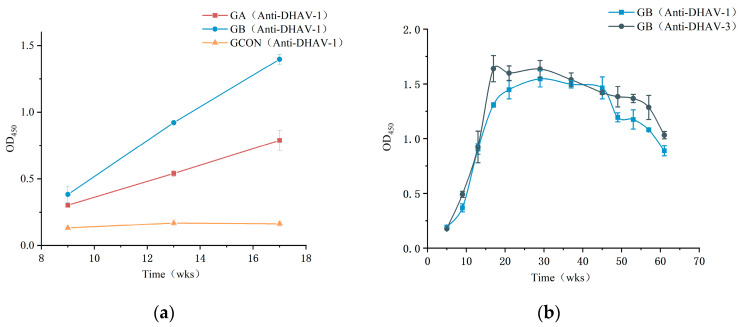
(**a**) Comparison of antibody titers in DHAV immunized layers using ELISA OD_450_ values. (**b**) Tracking titer of group B immunized layers further, using ELISA OD_450_ values. Error bars represent standard deviation (SD) of data (n = 3). Group A (GA) was vaccinated with Duck Viral Hepatitis Attenuated Vaccine, Living (Strain CH60). Group B (GB) received Duck Viral Hepatitis Bivalent (Serotype 1 + Serotype 3) Vaccine, Inactivated (Strain YB3 + Strain GD).

The injection of different immunogens into layers had a significant impact on the outcome variable at different time points, as shown by the interaction effect between time and group being significant (*p* < 0.05) in the repeated-measure ANOVA. This finding aligns with the experimental objective of identifying the most effective immunogen for IgY production in laying hens. As shown in Figure 1a, both the GA and GB groups produced IgY with significant activity. From the third to the fifth immunization, the antibody titers in both groups exhibited a gradual upward trend, with the GB group showing significantly higher IgY titers compared to the GA group. In contrast, the antibody titers in the GCON group remained consistently low. Based on these comparative results, the IgY produced in the GB group was selected for further experiments.

Antibody titers assessed at a concentration of 2 mg/mL and diluted 1:1000 began to increase one week after the second immunization, reaching their peak at week 17 for DHAV-3 and week 29 for DHAV-1. Although titers gradually declined by week 49, they remained notably high (OD_450_ > 1.0) at week 57, demonstrating that the immunization regimen successfully induced and maintained elevated antibody levels over an extended period (Figure 1b). Statistical analysis confirmed a significant effect of time on antibody titers, indicating dynamic changes throughout the study period (*p* < 0.05). These changes were particularly evident in the weeks following immunization.

### 3.2. IgY Neutralizing Activity by Virus Neutralization Test

At peak titer, IgY was collected, and its NI was assessed through a VN assay. Table 1 and Table 2 present the mortality rates for anti-DHAV-1 and anti-DHAV-3 IgY antibodies, measured by incubating a fixed concentration of antibody with various dilutions of the virus. The NI values were calculated using the Reed–Muench method, yielding indices of 38.90 against DHAV-1 and 141.25 against DHAV-3. The viral titers used were 10^6.33^ ELD_50_/mL for DHAV-1 and 10^7^ ELD_50_/mL for DHAV-3. The results showed that DHAV-1 and DHAV-3 incubated with anti-DHAV IgY exhibited reduced lethality compared to untreated controls, with mortality rates decreasing across the tested virus dilutions. Additionally, all duck embryos in the blank control group (injected with physiological saline) survived, underscoring the specificity and protective efficacy of anti-DHAV IgY.

### 3.3. IgY Effectiveness for DHAV-Infected Ducklings

#### 3.3.1. Mortality and Survival Rates

**Figure 2:**
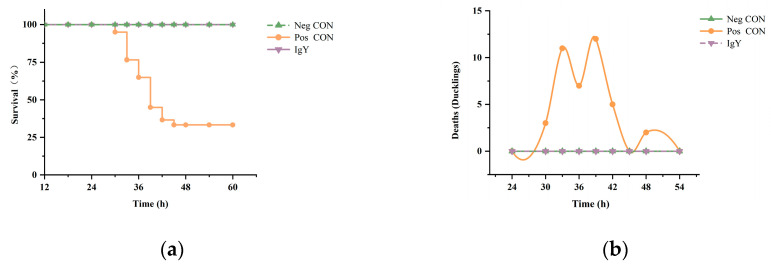
The effect of IgY on mortality in DHAV-infected ducklings. (**a**) The impact of IgY on the survival rate of DHAV-infected ducklings. The survival rates after treatment in the Neg CON group, Pos CON group, and IgY treatment group are shown (n = 60). (**b**) The effect of IgY on the number of ducklings succumbing to DHAV infection. The number of deaths among DHAV-infected ducklings at different time points after treatment in the Neg CON group, Pos CON group, and IgY treatment group (n = 60) is displayed.

Figure 2 illustrates the changes in survival rates and mortality of ducklings infected with DHAV-1 and DHAV-3 following IgY treatment. After IgY treatment, the survival rate in the IgY group reached 100%, comparable to that of the Neg CON group. In contrast, the Pos CON group showed a survival rate of 33%, significantly lower than that of the IgY treatment group. The number of mortalities in the Pos CON group generally exhibited an initial increase, reaching a peak between 36 and 42 hpi, followed by a gradual decrease. All mortality events occurred within the first 48 hpi.

#### 3.3.2. Histopathological Examination

**Figure 3:**
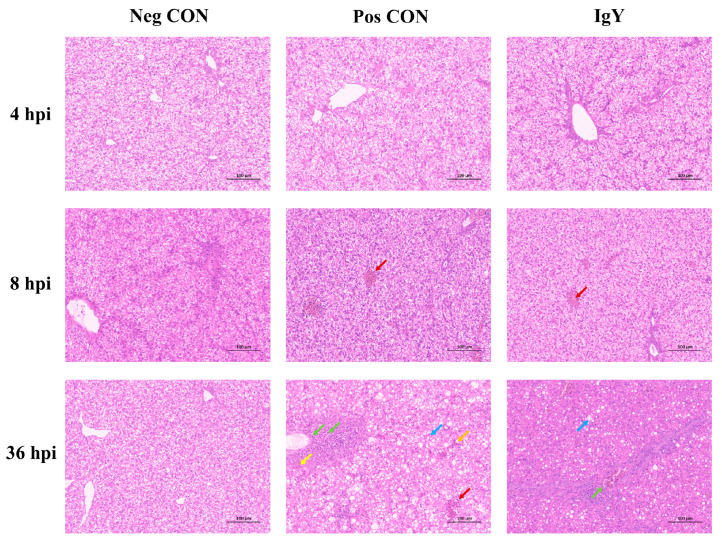
The impact of IgY on liver tissue morphology in ducklings from three different groups (Neg CON, Pos CON, and IgY) was examined using hematoxylin and eosin (H&E) staining. Images were captured at 200× magnification, with a scale bar included for reference.

The impact of IgY on liver tissue morphology in ducklings was assessed across three different groups: Neg CON, Pos CON, and IgY (Figure 3). In the Neg CON group, liver tissues maintained a normal structure and cellular arrangement throughout the study, showing no evident lesions or inflammatory cell infiltration. In the Pos CON group, no significant lesions were observed at the early time point (4 hpi). However, by 8 hpi and 36 hpi, the liver tissues exhibited progressive pathological changes, including vascular stasis (red arrow), fatty degeneration of hepatocytes (blue arrow), and cytoplasmic vacuoles. Additionally, there was infiltration of lymphocytes and granulocytes around blood vessels (green arrow), as well as the presence of basophilic cell aggregates (yellow arrow), occasional perivascular hemorrhages (red arrow), and dilated hepatic sinusoids with bruising (orange arrow). In the IgY group, liver tissue structure remained normal at 4 hpi, with no significant lesions observed. By 8 hpi, while there was increased perivascular hemorrhages (red arrow), no significant inflammatory cell infiltration was noted. At 36 hpi, despite some degree of steatosis (blue arrow), inflammatory cell infiltration was minimal, indicating relatively mild lesions (green arrow).

**Figure 4:**
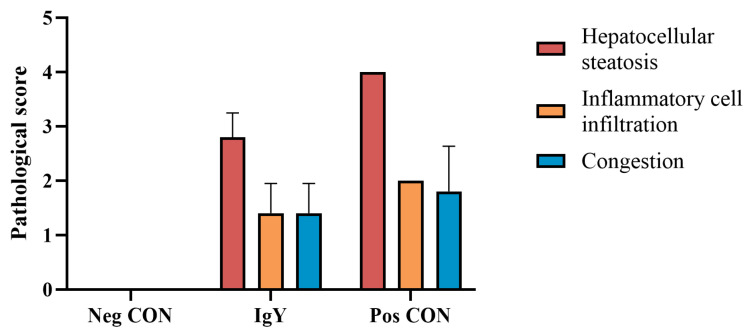
Histopathological scoring of liver tissue in ducklings.

Histopathological examination of the liver tissue in ducklings was conducted to observe the effects of egg yolk antibodies against DHAV on the liver tissue pathology of DHAV-challenged ducklings 36 h post-infection. The histopathological scores of the liver tissue in ducklings are shown in Figure 4. As indicated by Figure 3 and Figure 4, following DHAV challenge, the liver tissue of ducklings exhibited varying degrees of hepatocellular fatty degeneration, inflammatory cell infiltration, and congestion. Compared to the control group, the treatment group had higher pathological scores, but no significant differences were observed (*p* = 0.28). The pathogenic challenge control group had the highest total pathological score, which was significantly different from the control group (*p* = 0.04).

#### 3.3.3. Evaluating Liver Enzymes and Immune Markers Post-Infection

Table 3 shows the biochemical indicators measured in serum samples collected at 36 hpi. No significant differences were observed among the groups in TP, ALB, GLB, or A/G levels; however, significant differences were found in AST, ALT, and ALP levels. The Pos CON group exhibited significantly higher AST and ALT levels than the Neg CON group, while the AST levels in the IgY group did not differ significantly from those in the Neg CON group. Similarly, for ALP levels, the IgY group showed a significant decrease compared to the Pos CON group, with no significant difference from the Neg CON group.

#### 3.3.4. Evaluating Oxidative Stress Response Post-Infection

Table 4 presents the antioxidant capacity indicators measured in serum samples collected 36 hpi. No significant differences were observed in T-AOC levels among the groups; however, significant differences were found in MDA, T-SOD, CAT, and GSH-PX levels. Compared to the Pos CON group, the IgY group exhibited significantly lower MDA levels, with no significant difference from the Neg CON group. Conversely, the T-SOD, CAT, and GSH-PX levels in the IgY group were significantly higher than those in the Pos CON group and showed no significant difference from the Neg CON group. Lastly, the results of this study underscore the multifaceted role of IgY prophylactic treatment in mitigating oxidative stress induced by DHAV-1 and DHAV-3 infections in ducklings.

## 4. Discussion

After DHAV-1 and DHAV-3 infections, the liver is the primary target of the virus, leading to inflammation, liver damage, and oxidative stress, which further impairs immune function [8,24]. We immunized hens to produce potent anti-DHAV antibodies. We subsequently evaluated the virus-neutralizing properties of the IgY isolated from the egg yolks from the hyper-immunized hens. This agent showed potential in reducing inflammatory responses and alleviating oxidative stress caused by DHAV infection in ducklings.

We employed ELISA to evaluate the antibody titer levels and their changes over time. The OD_450_ values obtained directly reflect the binding efficacy of IgY to the virus. The adjuvanted Duck Viral Hepatitis Bivalent Vaccine (Serotype 1 + Serotype 3, Strain YB3 + Strain GD) resulted in significantly higher IgY titers, with an OD_450_ difference of approximately 0.6 compared to the antibodies produced by the Duck Viral Hepatitis Attenuated Vaccine, Living (Strain CH60). This increase aligns with the expected immune-enhancing effect of adjuvants (Figure 1a) [25]. Such a prolonged antibody response is crucial for generating IgY capable of protecting ducks from DHAV infection (Figure 1b). Egg yolk antibody was evaluated using a virus neutralization assay. As shown in Table 1 and Table 2, the results demonstrate the effective neutralizing capacity of IgY. This high specificity in binding is attributed to the unique immune recognition structure of IgY, which enables precise epitope recognition and high-affinity binding, further enhancing its prophylactic efficacy. Compared to IgG, IgY exhibits distinct structural and functional differences that influence its roles in immunity and applications. Structurally, IgY lacks the Fc region and hinge structure present in IgG, which reduces its ability to bind Fc receptors and activate the complement system in mammalian hosts [26]. Functionally, IgY focuses on antigen neutralization rather than immune effector functions, making it less inflammatory and more suitable for passive immunization [27]. Previous studies, such as that by El-Kafrawy et al., have shown that SARS-CoV-2-specific IgY can specifically bind to the receptor-binding domain of the virus, preventing its interaction with receptors on the host–cell surface [28]. The IgY antibodies likely exert their protective effect by specifically recognizing and binding to DHAV viral particles, thereby preventing their attachment to host–cell receptors and inhibiting viral entry and replication in host cells [29]. Moreover, IgY exhibits broad-spectrum antimicrobial activity, demonstrating significant inhibitory effects against bacteria and other viruses. Through careful antigen design and epitope selection, the broad-spectrum adaptability of IgY can be further enhanced, allowing it to effectively address the complex and diverse pathogen profiles encountered in livestock farming. This combination of specificity and broad-spectrum activity makes IgY an indispensable tool in pathogen control.

In general, the effectiveness of antiviral activity in vitro stems from the direct impact of antibodies on the virus. However, the in vivo environment is relatively more complex. To investigate the potential protective effects of IgY on ducklings infected with DHAV-1 and DHAV-3, a duckling liver inflammation model induced by these viruses was established [30]. In this model, the survival rate of the IgY group was significantly higher than that of the Pos CON group (Figure 2), indicating the potential prophylactic effect of the antibody against DHAV. To further validate this observation, we measured several biochemical markers commonly used to assess liver damage. We observed that in the IgY-treated group, AST, ALT, and ALP levels showed no significant difference compared to the Neg CON group, suggesting that IgY therapy may mitigate hepatocellular damage induced by DHAV-1 and DHAV-3. Histopathological analysis of liver tissues further supports this finding, demonstrating that specific IgY can alleviate DHAV-induced liver injury. The neutralizing capacity of IgY may reduce virus-induced hepatocellular damage, thereby lowering AST and ALT levels in the blood accordingly. Additionally, no significant differences were observed in TP, ALB, GLB, or the A/G ratio between the IgY group and the Pos CON group, possibly because these markers reflect liver synthesis and immune function rather than acute hepatocellular injury. Liver diseases, whether metabolic, degenerative, or proliferative in nature, are correlated frequently with oxidative stress [31]. This oxidative stress is recognized as a prevalent pathological mechanism in liver injury and is intricately connected to the progression of liver damage [32]. This study demonstrated that IgY treatment significantly enhances antioxidant defense mechanisms in DHAV-infected ducklings. As shown in Table 4, GSH-Px, CAT, and SOD activities were markedly elevated following IgY administration, while MDA levels, an indicator of lipid peroxidation, significantly decreased. These findings suggest that IgY treatment effectively reduces oxidative damage and protects against DHAV-induced hepatocellular injury. Histological analysis further revealed that liver tissues in the IgY group exhibited milder lesions compared to the Pos CON group at 36 hpi. These observations support the conclusion that IgY mitigates oxidative stress and inflammation in DHAV-infected ducklings.

These findings imply that IgY treatment effectively shields ducklings from oxidative damage induced by DHAV infection. The results suggest that IgY treatment may work by stimulating endogenous antioxidant pathways in the host. This stimulation involves elevating the activity of crucial antioxidant enzymes like GSH-Px, CAT, and SOD, which help eliminate excess free radicals and peroxides, thereby safeguarding cells from oxidative harm [33,34]. This reduction in MDA levels observed in the IgY treatment group may be attributed to the heightened activity of the antioxidant enzyme system due to IgY treatment. This increased enzyme activity diminishes the impact of free radicals on cell membrane lipids, thereby preserving the integrity and functionality of the cell membrane [35]. These findings are consistent with results of other researchers who have demonstrated the effectiveness of therapeutic interventions by assessing oxidative stress responses in ducklings post-infection [36,37,38]. Specifically, in the context of DHAV infection, the IgY prepared in this study showed strong specificity that significantly reduced oxidative stress and liver damage caused by DHAV. Notably, Chen et al. and Cao et al. found that DHAV infection correlates with decreased antioxidant enzyme levels and elevated liver injury markers (AST, ALT) [30,39], supporting the role of oxidative imbalance in DHAV-induced pathology [5]. Consistent with these findings, our results further confirm the multiple roles of IgY in protecting host–cell function. It not only inhibits viral replication but also promotes tissue repair by modulating host metabolism and immune responses.

Building on its ability to alleviate oxidative stress and mitigate tissue damage caused by DHAV, IgY also plays a crucial role in immune prevention and therapy in ducks and similar hosts through specific antigen binding, complement activation, and modulation of immune cell functions. Choi et al. demonstrated that IgY effectively neutralizes viruses and inhibits their infectivity by recognizing and binding to antigenic epitopes via its Fab fragment, thereby blocking the critical functions of pathogenic molecules [40]. On the other hand, in avian immune systems, IgY can form immune complexes that trigger activation of the complement system through both the classical and alternative pathways. This species-specific mechanism enhances pathogen clearance and reflects the distinct immune pathways in avians [41]. Furthermore, IgY-tagged pathogens are more efficiently phagocytosed by macrophages, while IgY also stimulates the proliferation of T and B lymphocytes, enhancing both cellular and humoral immune responses [42]. Collectively, these mechanisms synergize to not only strengthen the host’s immune defenses but also enhance the scientific rigor and efficacy of therapeutic interventions through precise cellular and molecular regulation. Compared to traditional vaccines, IgY offers the added advantage of providing immediate protection, making it particularly useful for emergency control of outbreak diseases. While vaccines require time to induce an immune response, IgY can exert passive immunity rapidly, providing protection to the host within a short time frame, thereby reducing mortality and the risk of disease transmission.

Egg yolk antibody as a safe, sustainable, and efficient biopharmaceutical, holds strategic significance in the prevention and control of livestock and poultry diseases. In the future, its application can be further expanded to the prevention and treatment of various infectious diseases. By integrating modern biotechnologies, such as genetic engineering and synthetic biology, the production of IgY can overcome existing technical limitations, enabling more efficient and precise antibody production. Additionally, IgY can serve as a core tool in animal health management, complementing vaccines and antibiotic alternatives to establish a comprehensive disease control system. In the context of increasing global attention to food safety and animal health, the widespread adoption and application of IgY are expected to significantly improve the health levels and economic efficiency of livestock farming.

## 5. Summary

Given the highly contagious nature and high lethality of DHAV, its control presents significant challenges, leading to substantial economic losses in the poultry industry. Our results show that IgY holds promise as a potential prophylactic and possibly therapeutic strategy which also address DHAV-1 and DHAV-3 infections and reduces duckling mortality. In this context, the prophylactic efficacy of IgY in ducklings infected with DHAV is particularly significant. The potential mechanisms may include direct virus neutralization, activation of antioxidant defense mechanisms, reduction in lipid peroxidation, and immunomodulation. These combined mechanisms work towards alleviating oxidative stress and cellular damage induced by DHAV infection and ultimately safeguard ducklings from the virus. This research and application of IgY technology offer a way for the development of antibody-based therapies for prophylactic and therapeutic treatment of livestock diseases.

## Figures and Tables

**Table 1:** Mortality and survival data for duck embryos inoculated with anti-DHAV-1 IgY antibodies by virus neutralization assay.

Virus Dilution	Deaths	Survivors	Cumulative Death	Cumulative Survivor	Mortality
10^3.03^	4	0	12	0	100.0%
10^3.33^	3	1	8	1	88.9%
10^3.63^	1	3	5	4	55.6%
10^3.93^	2	2	4	6	40.0%
10^4.23^	1	3	2	9	18.2%
10^4.53^	1	3	1	12	7.7%

**Table 2:** Mortality and survival data for duck embryos inoculated with anti-DHAV-3 IgY antibodies by virus neutralization assay.

Virus Dilution	Death	Survivor	Cumulative Death	Cumulative Survivors	Mortality
10^2.70^	3	1	9	1	90.0%
10^3.40^	2	2	6	3	66.7%
10^4^	2	2	4	5	44.4%
10^4.6^	1	3	2	8	20.0%
10^5.2^	1	3	1	11	8.3%

**Table 3:** Biochemical indicators in DHAV-infected ducklings with and without IgY treatment compared to negative control group (mean ± 95% CI).

Biochemical Indicators	Neg CON	95% CI (Neg CON)	Pos CON	95% CI (Pos CON)	IgY	95% CI (IgY)	*p*-Value
TP (g/L)	20.14	(17.63, 23.50)	19.54	(18.59, 20.59)	20.69	(17.06, 22.42)	0.647
ALB (g/L)	8.31	(6.76, 9.73)	8.23	(7.60, 8.77)	9.24	(8.42, 9.95)	0.146
GLB (g/L)	11.53	(8.57, 14.14)	11.335	(10.45, 12.66)	12.11	(10.41, 13.46)	0.731
A/G	0.68	(0.66, 0.69)	0.71	(0.60, 0.79)	0.68	(0.61, 0.75)	0.676
AST (U/L)	38.63 ^b^	(18.00, 83.40)	164.05 ^a^	(42.29, 309.70)	51.65 ^ab^	(38.70, 73.90)	0.038
ALT (U/L)	20.68 ^b^	(17.47, 27.71)	64.08 ^a^	(37.02, 87.52)	35.25 ^b^	(14.58, 50.63)	0.002
ALP (U/L)	892.21 ^ab^	(796.67, 1104.40)	1094.45 ^a^	(843.05, 1279.02)	751.74 ^b^	(587.92, 866.45)	0.009

Note: for a, b, values with different superscripts within a row differ (*p* < 0.05), n = 5. Neg CON refers to the negative control group (no virus and no IgY), Pos CON represents the positive control group (challenged with DHAV), and IgY indicates the experimental group (challenged with DHAV and treated with IgY).

**Table 4:** Oxidative stress indicators in DHAV-infected ducklings with and without IgY treatment compared to a negative control group (mean ± 95% CI).

Indicators of Oxidative Stress	Neg CON	95% CI (Neg CON)	Pos CON	95% CI (Pos CON)	IgY	95% CI (IgY)	*p*-Value
MDA (nmol/mg)	4.18 ^b^	(3.23, 4.76)	5.31 ^a^	(4.09, 5.98)	3.51 ^b^	(3.11, 3.84)	0.001
T-SOD (U/mL)	115.81 ^a^	(108.05, 128.30)	92.35 ^b^	(80.19, 107.20)	142.49 ^a^	(129.15, 169.67)	<0.001
CAT (U/mL)	7.18 ^ab^	(6.14, 8.27)	5.19 ^b^	(4.29, 6.59)	8.74 ^a^	(6.82, 10.7)	0.003
GSH-PX (U/mL)	275.60 ^a^	(250.55, 291.43)	250.29 ^b^	(228.13, 267.69)	297.76 ^a^	(283.52, 312.53)	<0.001
T-AOC (mmol/L)	0.38	(0.24, 0.54)	0.30	(0.19, 0.38)	0.39	(0.31, 0.46)	0.239

Note: for a, b, values with different superscripts within a row differ (*p* < 0.05), n = 5. Neg CON refers to the negative control group (no virus and no IgY), Pos CON represents the positive control group (challenged with DHAV), and IgY indicates the experimental group (challenged with DHAV and treated with IgY).

## Data Availability

The data presented in this study are available upon request from the corresponding author. However, the data are not publicly available due to privacy concerns.
